# The FIGO Ovulatory Disorders Classification System^†^

**DOI:** 10.1093/humrep/deac180

**Published:** 2022-08-19

**Authors:** Malcolm G Munro, Adam H Balen, SiHyun Cho, Hilary O D Critchley, Ivonne Díaz, Rui Ferriani, Laurie Henry, Edgar Mocanu, Zephne M van der Spuy, FIGO Committee on Menstrual Disorders and Related Health Impacts, and FIGO Committee on Reproductive Medicine, Endocrinology, and Infertility, Ganesh Acharya, Georgios Adonakis, Sadiah Ahsan, Taghreed AIhaidari, Tengiz Asatiani, Ricardo Azziz, Adam Balen, Michela Bedard, Jennifer Blake, Veronica Chamy, Ying Cheong, Vincent Y T Cheung, Si Hyun Cho, Hilary Critchley, Jose Teixeira da Silva, Ivonne Diaz, Colin Duncan, Amelie Ekersley, Roberto Epifanio-Malpassii, Abimbola Famuyide, Rui Ferriani, Linda Giudice, Maargarita Gurevich, Sioban Harlow, Roger Hart, Oskari Heikinheimo, Laurie Henry, Sulaiman Heylen, Richard Kennedy, Anna Klepchuckova, Petr Krepelka, Paul le Roux, Kateryna Levchenko, Dimitrios Loutradis, Erica Marsh, Noni Martins, Raj Mathur, Thabo Matsaseng, Rui Miguelote, Edgar Mocanu, Malcolm Munro, Eugene Ngoga, Michelle Nisolle, Robert Norman, Masanori Ono, Axelle Pintiaux, Gunda Pristauz-Telsnigg, Diana Ramasauskaite, Pernille Ravn, Jose Reis, Peter Roos, Irena Rozic, Anibal Scarella, Katsiaryna Sharai, Alena Shibut, Sony Sierra, Anne Steiner, Dominic Stoop, Bettina Toth, Zephne Van Der Spuy, Saskia Williams, Lauren Wise, Anusch Yazdani, Liudmila Zhaunova, Meggan Zunckel, Karabo Zwane

**Affiliations:** The University of California, Los Angeles, Los Angeles, CA, USA; The University of Leeds, Leeds, UK; Yonsei University, Seoul, South Korea; The University of Edinburgh, Edinburgh, UK; Nueva Granada University, Bogota, Colombia; The University of São Paolo, São Paolo, Brazil; Centre Hospitalier Universitaire Liège, University of Liège, Liège, Belgium; Trinity College, Dublin, Ireland; The University of Cape Town, Cape Town, South Africa

**Keywords:** anovulation, ovulatory disorders, ovulatory disorders classification, ovulatory dysfunction

## Abstract

Ovulatory disorders are common causes of amenorrhea, abnormal uterine bleeding and infertility and are frequent manifestations of polycystic ovary syndrome (PCOS). There are many potential causes and contributors to ovulatory dysfunction that challenge clinicians, trainees, educators, and those who perform basic, translational, clinical and epidemiological research. Similarly, therapeutic approaches to ovulatory dysfunction potentially involve a spectrum of lifestyle, psychological, medical and procedural interventions. Collaborative research, effective education and consistent clinical care remain challenged by the absence of a consensus comprehensive system for classification of these disorders. The existing and complex system, attributed to the World Health Organization (WHO), was developed more than three decades ago and did not consider more than 30 years of research into these disorders in addition to technical advances in imaging and endocrinology. This article describes the development of a new classification of ovulatory disorders performed under the aegis of the International Federation of Gynecology and Obstetrics (FIGO) and conducted using a rigorously applied Delphi process. The stakeholder organizations and individuals who participated in this process comprised specialty journals, experts at large, national, specialty obstetrical and gynecological societies, and informed lay representatives. After two face-to-face meetings and five Delphi rounds, the result is a three-level multi-tiered system. The system is applied after a preliminary assessment identifies the presence of an ovulatory disorder. The primary level of the system is based on an anatomic model (Hypothalamus, Pituitary, Ovary) that is completed with a separate category for PCOS. This core component of the system is easily remembered using the acronym HyPO-P. Each anatomic category is stratified in the second layer of the system to provide granularity for investigators, clinicians and trainees using the ‘GAIN-FIT-PIE’ mnemonic (Genetic, Autoimmune, Iatrogenic, Neoplasm; Functional, Infectious and Inflammatory, Trauma and Vascular; Physiological, Idiopathic, Endocrine). The tertiary level allows for specific diagnostic entities. It is anticipated that, if widely adopted, this system will facilitate education, clinical care and the design and interpretation of research in a fashion that better informs progress in this field. Integral to the deployment of this system is a periodic process of reevaluation and appropriate revision, reflecting an improved understanding of this collection of disorders.

Glossary of terms used in this article

**Table:** 

Abnormal uterine bleeding (AUB)	Implicitly, non-gestational and in the reproductive years. Any alteration in the normal frequency, regularity, duration or volume of menstrual bleeding (including HMB) as well as intermenstrual bleeding and unscheduled bleeding with pharmaceutical agents designed to suppress menstrual function
Acute heavy menstrual bleeding	An episode of HMB of sufficient volume to require immediate therapy
Amenorrhea	A symptom—absence of menstrual bleeding in a girl or woman in the reproductive years
Anovulation	Failure to ovulate
Chronic abnormal uterine bleeding (AUB)	Symptoms of AUB for the majority of the past 6 months
Chronic ovulatory disorder	Evidence of an ovulatory disorder for the majority of the previous 6 months
Frequent menstruation	An AUB symptom—menstrual cycle of less than 24 days
Heavy menstrual bleeding (HMB)	An AUB symptom—excessive menstrual blood loss that interferes with a woman’s physical, social, emotional and/or material quality of life
Infrequent menstruation	An AUB symptom—menstrual cycle length of more than 38 days
Intermenstrual bleeding	An AUB symptom—uterine bleeding between regular menstrual periods
Irregular menstruation	An AUB symptom—menstrual cycle lengths that vary by more than 7 (ages of 18–25 and 42–45 years) to 9 days (ages of 26–41 years)
Luteinized unruptured follicle (LUF)	Physical failure of follicle rupture (oocyte release), with the luteinization and other endocrine features of the secretory phase of the menstrual cycle
Luteal out of phase (LOOP) event	Premature recruitment of a follicle in the luteal phase of a menstrual cycle
Menstrual cycle	The duration in days from the first day of one menstrual period to the first day of the next
Ovulation	The release of an oocyte (egg) from an ovarian follicle
Ovulatory disorder	Any alteration of ovulatory function in non-pregnant women in the usual reproductive years
Primary amenorrhea	Failure of onset of menstruation by the age of 15 years
Prolonged menstruation	An AUB symptom—a menstrual period lasting more than 8 days
Secondary amenorrhea	Absence of menstrual periods for more than 180 days in an individual who has had at least one spontaneous menses

## Introduction

Ovulatory disorders are common in girls and women of reproductive age and are associated with episodic or chronic dysfunction of the hypothalamic–pituitary–ovarian axis (Hickey and Balen, 2003; Teede *et al.*, 2018). These disorders may adversely affect quality of life when they manifest with infertility or as aberrations in menstrual function. Menstrual symptoms may include altered frequency or regularity of flow, as well as prolonged or heavy menstrual bleeding (HMB), or even a complete absence of menstrual blood flow, referred to as amenorrhea (Munro *et al.*, 2018). Reproductive function may be adversely impacted as chronic anovulation is a common cause of infertility. While there are numerous known causes and contributors to ovulatory disorders, the entire spectrum of mechanisms of pathogenesis remains to be fully elucidated. Ovulatory disorders are often associated with underlying endocrinopathies, neoplasms, psychological and psychiatric conditions, and the use of specific pharmacologic agents. Optimally effective research, teaching and clinical management of ovulatory disorders have been impeded by the absence of a comprehensive, internationally recognized and utilized structured classification system.

The World Health Organization (WHO) system for ovulatory disorders was first presented as a monograph in 1973 (WHO-Scientific-Group, 1973) and has been modified over time in various reviews and book chapters by single authors rather than international consensus. Some 50 years later, much more is known about ovulatory disorders. As a result, the International Federation of Gynecology and Obstetrics (FIGO) has undertaken a process whereby the global community of stakeholders involved with ovulatory disorders has designed a new system to better meet the needs of investigators, clinicians and medical educators worldwide. The development of the system started with the formation of an Ovulatory Disorders Steering Committee (ODSC) comprising members of FIGO’s Committee on Menstrual Disorders (MDC) (now the Committee on Menstrual Disorders and Related Health Impacts, or MDRHI) and Committee on Reproductive Medicine, Endocrinology, and Infertility. The involvement of the MDRHI reflects the common and important impact of ovulatory disorders on menstrual bleeding experience, an entity referred to as AUB-O in FIGO System 2 (see below).

## Background and rationale

### Defining ovulatory disorders

In the reproductive years—and in the absence of pregnancy, the process of lactation or the use of pharmacological agents such as contraceptive steroids—the normal woman releases a mature oocyte from a Graafian follicle in a relatively predictable and cyclical fashion. However, a consensus definition of ovulatory disorders, sometimes called ovulatory dysfunction, has been lacking. The notion of anovulation or absent ovulation is but one manifestation, but there exists a spectrum of chronic or episodic conditions or circumstances that also disrupt the predictable and cyclical ovulatory process. Previously, infrequent ovulation has been termed ‘oligo-ovulation’, which typically, but not always, manifests with some combination of infrequent and irregular onset of menstruation as defined in FIGO AUB System 1 (FIGO discontinued the term oligomenorrhea). However, and recognizing that many women with ovulatory disorders may have normal-length menstrual cycles (Prior *et al.*, 2015), no clear definition of infrequent ovulation has been adopted and was not addressed in the joint ‘Committee Opinion’ on Infertility Workup for the Women’s Health Specialist produced by the American College of Obstetricians and Gynecologists and the American Fertility Society (ACOG, 2019).

Furthermore, while an occasional failure to ovulate is expected and may not contribute to infertility, it may well cause an episode of delayed onset of menses and even HMB. This circumstance begs the inclusion of intermittent anovulation in a broad-based, all-encompassing definition of ovarian dysfunction. An additional consideration is other aberrations in ovulatory function, such as the luteinized unruptured follicle (LUF) (Bashir *et al.*, 2018; Li *et al.*, 2021) and the luteal out of phase (LOOP) events (Hale *et al.*, 2009) that represent, respectively, mechanical failure to release the mature oocyte and the premature recruitment of follicles in the luteal phase, each of which could be candidates for inclusion in the definition of ovulatory dysfunction.

As a result of these considerations, it is apparent that there is an unmet need for both a revised definition of ovulatory disorders and a consensus classification system designed to guide research, education and clinical care across disciplines.

### Existing ‘system’ and its value and limitations

The original WHO classification presented three types of ovulatory dysfunction (WHO-Scientific-Group, 1973).

Group I included ‘women with amenorrhea and with little or no evidence of endogenous estrogen activity, including patients with (a) hypogonadotrophic ovarian failure, (b) complete or partial hypopituitarism or (c) pituitary–hypothalamic dysfunction’. Group II was described as ‘Women with a variety of menstrual cycle disturbances (including amenorrhea) who exhibit distinct estrogen activity (urinary estrogens usually <10 µg/24 h), whose urinary and serum gonadotrophins are in the normal range and fluctuating, and who may also have fairly regular spontaneous menstrual bleeds (i.e. 24–38 days apart) but without ovulation’. Group III was described as ‘Females with primary ovarian failure (sic, now known as primary ovarian insufficiency; POI) associated with low endogenous estrogen activity and pathologically elevated serum and urinary gonadotrophins’. This classification illustrates the now-outdated assay methodology of the time.

A second monograph was published in 1976, which presented an algorithm based upon whether the serum prolactin concentration was elevated or normal, the response to a progestagen challenge test to assess estrogenization, and whether the serum FSH concentration was elevated or normal (WHO-Scientific-Group, 1976). The results of these assays were to be used to define seven groups:

- Group I: Hypothalamic pituitary failure
- Group II: Hypothalamic pituitary dysfunction
- Group III: Ovarian failure
- Group IV: Congenital or acquired genital tract disorders
- Group V: Hyperprolactinemia, with a space-occupying lesion
- Group VI: Hyperprolactinemia, with no detectable space-occupying lesion
- Group VII: Non-functioning hypothalamic/pituitary tumors (WHO-Scientific-Group, 1976)

Over the last 40 years, numerous descriptions of the WHO classification have appeared in various monographs and book chapters in textbooks on gynecology, infertility and reproductive endocrinology. Multiple authors have modified the classification without any evidence of further scientific discussion or consensus development. Interestingly, the UK NICE Guidelines on the investigation and management of infertility, first published in 2004 (NICE, 2013), describe three groups with reference to the *WHO Manual for the Standardized Investigation and Diagnosis of the Infertile Couple*, published in 1993 (Rowe *et al.*, 1993). Yet this WHO manual does not contain any classification of ovulatory disorders. Nonetheless, the NICE classification (NICE, 2013) encompasses the three groups that most authors refer to currently, namely:

- Group I: Low gonadotropins and estradiol
- Group II: ‘Gonadotropin disorder’ and normal estradiol
- Group III: High gonadotropins and low estradiol

In this classification, Group I essentially refers to hypogonadotropic hypogonadism and pituitary insufficiency but also includes hyperprolactinemia. Group II is often referred to as ‘hypothalamic/pituitary dysfunction’, and most consider this group to primarily comprise women with polycystic ovary syndrome (PCOS) (Teede *et al.*, 2018), while Group III is consistently POI. However, it is essential to appreciate that hormone levels do not obey clear rules. For example, in those with hypothalamic amenorrhea who are underweight, levels of serum LH are usually suppressed, while levels of FSH are often in the normal range (Frisch, 1987; Morrison *et al.*, 2021). In addition, women with PCOS often have levels of FSH and LH in the normal range (Balen, 2004). Furthermore, anovulation is only one extreme of ovulatory dysfunction that includes a spectrum of manifestations that range from isolated episodes to chronic ovulatory failure.

Since the first iterations of the WHO classification, there have been significant advances in understanding the control of ovulation and the pathophysiology of ovulatory disorders, together with improvements in assay technology and genomics. Consequently, there exists a need for a more comprehensive and updated classification.

### The FIGO systems for abnormal uterine bleeding in the reproductive years

In 2011 (Munro *et al.*, 2011a), and again in 2018 (Munro *et al.*, 2018), FIGO published its two systems for describing nongestational abnormal uterine bleeding (AUB) in the reproductive years, including System 2, the classification system known as ‘PALM-COEIN’ that categorizes causes of AUB in non-gravid women of reproductive age, including those with ovulatory disorders (AUB-O). These systems were developed and designed using a rigorous Delphi process, with the participants including international experts and representation from multiple and diverse stakeholder organizations, including national and subspecialty societies and journals and the US Food and Drug Administration. The overall process also included an examination of the available population databases dealing with menstruation that resulted in new, evidence-based definitions for normal and abnormal menstrual metrics that are now known as the FIGO AUB System 1 (Fraser *et al.*, 2007a; Munro *et al.*, 2018). The process has been iterative with periodic revisions of systems that reside in what is described as a ‘living document’. The whole process has been underpinned and continues to be supported by FIGO and the FIGO Committee on Menstrual Disorders (MDC), which, since 2022, has been known as the Committee on Menstrual Disorders and Related Health Impacts.

FIGO AUB System 1 describes non-gestational normal and AUB in the reproductive years and addresses the features of menstruation, that is, frequency, regularity, duration and perceived volume of menstrual blood loss in addition to the presence of bleeding between periods (intermenstrual bleeding) as well as unscheduled bleeding associated with the use of gonadal steroids for contraception (Munro *et al.*, 2018). The latter is now encompassed by the increasingly used term ‘contraceptive-induced menstrual bleeding changes’ (CiMBC) (Polis *et al.*, 2018). Notably, System 1 is currently based upon data from studies of women aged 18–45 years, as evidence from adolescent girls and women in the late reproductive years is less well defined.

The second system, FIGO AUB System 2, describes potential causes or contributors to symptoms of AUB that are categorized in System 1 (Munro *et al.*, 2018). The nine categories, arranged according to the acronym PALM-COEIN, are as follows: Polyp (AUB-P); Adenomyosis (AUB-A); Leiomyoma (AUB-L); Malignancy and hyperplasia (AUB-M); Coagulopathy (AUB-C); Ovulatory dysfunction (AUB-O); Endometrial disorders (AUB-E); Iatrogenic (AUB-I); and Not otherwise classified (AUB-N). For the present context, ovulatory disorders (AUB-O) incorporate a range of disturbances in normal ovulatory function ranging from irregular to infrequent to absent ovulation. To date, in the context of management of patients with AUB, the diagnosis of ovulatory disorders has been based mainly on a detailed menstrual history to meet the parameters that comprise FIGO System 1. In the 2018 revisions of the two FIGO systems, the recommendation was made that treatments that may interfere with the H-P-O axis and associated with AUB be placed within the ‘AUB-I’ category (Munro *et al.*, 2018). The rationale and methodology for developing a sub-classification system for AUB-O are now presented.

## Methodology

The approach selected was based on RAND Delphi methodology, extensively used for consensus development processes, including classification systems for medical conditions (RAND, 2020). The two FIGO systems for AUB in the reproductive years, the sub-classification systems for leiomyomas (AUB-L) and adenomyosis (AUB-A), now undergoing validation, have all been developed using a version of this process (Fraser *et al.*, 2007b; Munro *et al.*, 2011b). The project was submitted to and approved by the FIGO Executive and FIGO’s Education Communication and Advocacy Consortium (ECAC) approved the results before submission of the manuscript.

### Ovulatory Disorders Steering Committee

The first step was to form an Ovulatory Disorders Steering Committee (ODSC) comprising members of FIGO’s MDC (now MDRHI) and Committee on Reproductive Medicine, Endocrinology, and Infertility. The chairs of each of these committees collaborated to form the ODSC by identifying eight members from their committees, adding an external member who had a leadership position in the Global PCOS Alliance. The resulting nine-member committee had diverse reach and comprised one from each of the continents of Africa, Asia and North America, and two from each of the European Union, the UK and South America. The ODSC met at regular intervals between June and December 2020 to identify and engage stakeholders and develop and test the consensus process. The scope of the ODSC also included review and analysis of the results of the various rounds and in the design and testing of subsequent Delphi rounds.

### Stakeholder and participant identification

The first task of the ODSC was to identify and engage the appropriate stakeholders necessary for the Delphi process. The chosen categories included the following:

1. National obstetrical and gynecological societies
2. Subspecialty societies representing reproductive endocrinologists
3. Specialty (obstetrics and gynecology) and subspecialty (reproductive endocrinology and infertility) journals
4. Recognized experts in ovulatory disorders not participating in categories 1–3
5. Lay organizations interested in infertility, AUB or PCOS

Descriptive letters were created and customized for the various categories describing the rationale for the process and a synopsis of the methodology. Via the FIGO record of member countries, each of the national obstetrical and gynecological societies was contacted and invited by email to support the process by naming a representative. The ODSC identified the spectrum of subspecialty societies on the six continents and contacted leadership to explain the process and solicit support. The descriptive letter was sent electronically to both the society headquarters and the identified participant. A similar process involved the editorial offices of relevant specialty and subspecialty journals. The ODSC then identified recognized experts based on a combination of personal knowledge of the field and a search of the literature, subtracting those identified by national societies, subspecialty societies or journals for representation. Finally, the ODSC sought to identify lay organizations that could represent women and adolescent girls who may have ovulatory disorders. These groups were generally contacted directly, and if there was interest and an indication of commitment, a lay-based version of the letter was sent.

### The Delphi consensus process

#### Background and scoring system

The Delphi process was developed by the RAND Corporation as a method for determining multi-stakeholder expert consensus in a semi-anonymous fashion that minimizes the impact of interpersonal issues on the outcome (RAND, 2020). Originally designed to forecast the impact of technology on warfare, it has subsequently been utilized across a number of disciplines including health care. Versions of the Delphi Process were used previously in the development of the FIGO AUB systems (Fraser *et al.*, 2007a,b; Munro *et al.*, 2011a,b) and are generally similar to the original RAND system comprising a series of survey rounds designed to be administered in a web-based or live environment with electronic scoring. Members of the ODSC did not participate in the Delphi process as participants. The scoring system has nine levels (1–9), with ‘1’ being the most substantial disagreement with a statement, ‘9’ the strongest agreement, and ‘5’ representing neutrality. Scores in the top tertile (7, 8 and 9) indicated ‘agreement’ with a statement, while those in the bottom tertile (1, 2 and 3) were indications of disagreement. As a result, the remaining scores (4, 5 and 6) comprised the ‘neutral’ category, with ‘4’ leaning to disagreement and ‘6’ leaning to agreement. The minimum requirement for consensus agreement was a mean score of at least 7 (scores of 6.5–6.9 were rounded to 7), with no more than 15% in the disagreement category. Conversely, ‘disagreement’ was defined as a mean score of 3 or less (scores of 3.1–3.4 were rounded to 3), with no more than 15% in the agreement category. For each statement or question in a survey, there is a field to allow for free-text comments by the participants.

#### Participant orientation meeting

Before distributing the first round of surveys, two orientation meetings for the participants were held to ensure that the appropriate contact information was in the study database and systems and that all understood the survey mechanisms. The two meetings were held on the Zoom platform (Zoom Video Communications Inc, San Jose, CA, USA), with dates and times selected to facilitate flexibility for the diverse group of participants, particularly considering the spectrum of world time zones involved. Included in the messaging of this meeting was the understanding that Delphi participant answers would remain confidential and that all distributions would be anonymized. Demonstrations of the functionality of the system were provided. A session was recorded and uploaded to an accessible server for individuals who could not attend either of the live, web-based meetings and to provide a resource for all participants who wished to review the instructions on their own time. It is to be noted that the lay component of the process was planned to occur after the medical stakeholders had developed a draft system.

#### Conduct of the first round

The first round of the Delphi process was designed to identify the participants’ age, gender, location, expertise and constituency and evaluate general opinions, the latter using statements intended to elicit an ‘agree’ or ‘disagree’ response. These statements were crafted in a fashion that invited and measured opinions regarding the clinical relevance of ovulatory disorders, the need for a well-designed classification system, and the broad categories that should be included if such a system was to be designed. The draft set of questions was created by the Chair of the ODSC, reviewed by the committee members in meetings using the Zoom platform, and then tested on the web-based survey instrument SurveyMonkey (Momentive, San Mateo, CA, USA).

The final version of the first round was distributed to the stakeholders via their identified email addresses within the web-based survey system. The ODSC Chair, who also functioned as the Facilitator, kept track of responses and sent out reminder emails at intervals of 7–10 days until there were no additional responses.

The data were then exported to an Excel (Microsoft Corp, Everett, WA, USA) workbook comprising spreadsheets containing the survey template that automatically calculated means and the percentage of answers in the agree (7–9), neutral (4–6) and disagree (1–3) categories. The free-text comments made by the participants were also included in the spreadsheet. The ODSC reviewed these data as a prelude to the design of the second round. The aggregate anonymized results were sent to each participant along with a copy of their responses for comparative purposes.

#### Conduct of the second round

The second-round survey was constructed, in part, based upon the first-round results. Some ‘neutral’ responses that had marginal scores close to 3 or 7, or defined principally by the outliers, were reviewed in particular because, in such circumstances, it was possible that rewording a question or providing appropriately representative evidence would result in a change in the participant’s opinion. It was also possible that ‘re-asking’ the question in the context of individual participant understanding of the group response might result in changes in individual responses. This information allowed the ODSC to construct a second survey round that eliminated items with defined agreement or disagreement but included reworded statements and new statements seeking to refine and expand the criteria that the participants thought necessary.

The distribution of the second-round survey was confined to those participating in and responding to the first round. The web-based system, distribution and follow-up reminder technique were again employed. The data were retrieved, exported into the same Excel workbook with worksheet templates and analyzed by the ODSC. Similarly, the participants received an anonymized summary of the participant responses to each of the items and a copy of their answers for comparison.

At this point, the committee had enough information to design a draft system that addressed and included the elements identified in the first two Delphi rounds. This was conducted iteratively until a draft acceptable to all ODSC members was created.

#### Conduct of the third round

As a prelude to the live stakeholder meeting, a short clarifying third round was created, tested, distributed and the results analyzed by the ODSC, conducted in a fashion similar to that of the first two rounds. Included in this round was a version of the draft system with solicitation of preliminary opinions from the participants. As was the case for the first two rounds, each participant was provided an anonymized copy of the results of the previous round and a copy of their responses, all for review before the live participant meeting.

#### Participant meeting

All medical participants and the ODSC were invited to participate in the stakeholder meeting held live on the Zoom platform. Here, the overall results of the survey rounds were presented, including those items where consensus one way or the other had not been reached. The draft system was also reviewed. An open discussion was invited, and preliminary polls were taken using the system available on the Zoom platform.

#### Post-meeting and fourth survey round

The ODSC undertook the post-meeting analysis. Subsequently, a short fourth-round poll was conducted to reach a consensus on the remaining elements and include individuals who could not participate in the live meeting.

#### Lay round

The lay round was designed to query the lay representatives, both for their perception of a need for a classification system and their opinions of the system developed by the expert and representative participants. A separate survey was designed that included some of the items in the medical participant rounds but presented in a fashion accessible by a lay audience. There was a focus on their opinions of clarity and utility in the context of discussion and counseling involving healthcare practitioners and patients. The draft lay-round elements were reviewed and revised by the ODSC, uploaded to the SurveyMonkey platform, tested and then distributed to the participants in a fashion similar to that used for the medical participant rounds. The results were reviewed and analyzed by the ODSC, who considered these opinions in revising the system and constructing the manuscript and the design of materials for the lay audience.

## Results

### Medical expert participants

**Figure 1.**
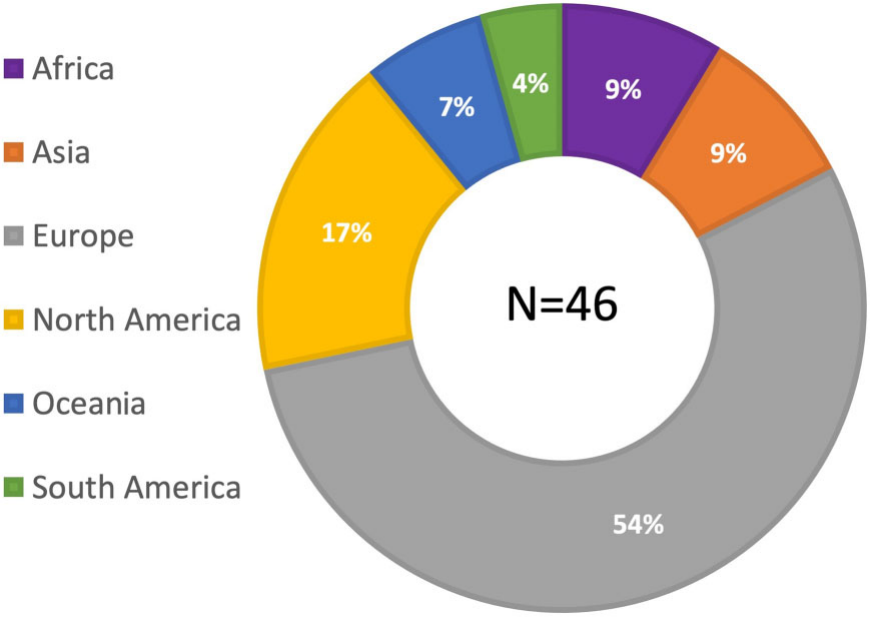
**Participants by region, displayed as a percentage.** *Note*: While there was representation from every region, Europeans comprised the majority.

**Figure 2.**
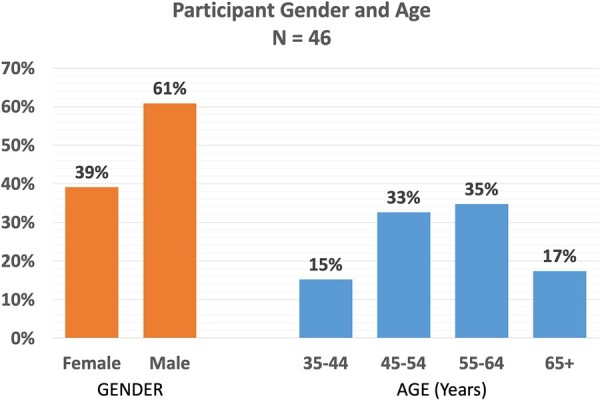
**Participants by age and gender.** *Note*: The proportion of men versus women and the age distribution are displayed.

**Figure 3.**
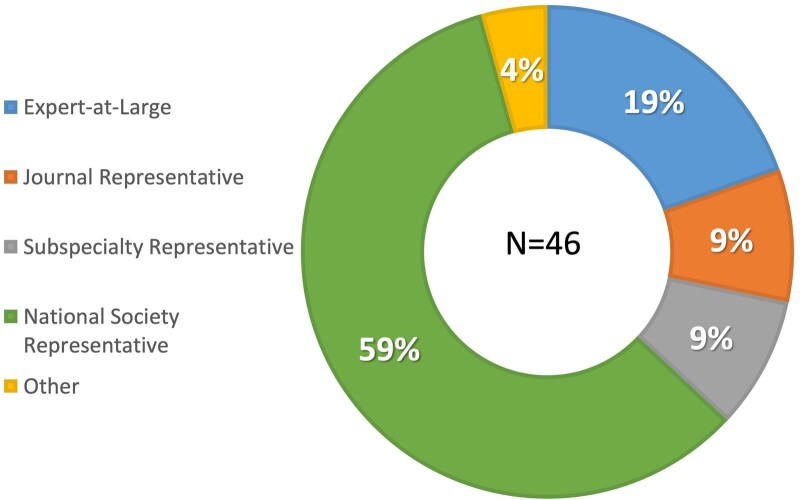
**Participants by stakeholder representation.** *Note*: Almost 60% of the participants represented national obstetrical and gynecological societies, while 19% were deemed ‘Experts at large’ based primarily on their contributions to the scientific literature. Journal and subspecialty representatives each comprised 9% of the participant pool.

**Figure 4.**
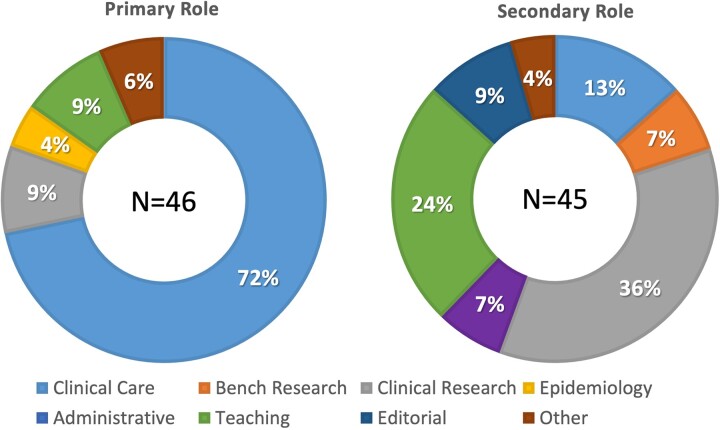
**Participants’ roles in their local institution or organization.** *Note*: Each participant was asked to reveal their primary (left) and secondary (right) roles or responsibilities in their local institution or organization. Almost three-quarters were primarily involved in clinical care, and there were no individuals who reported that bench research or editorial activity was their primary role. More than one-third saw clinical research as their secondary role, while almost one-quarter reported teaching as their secondary responsibility.

A total of 88 invitations were sent to the responding national gynecological and obstetrical societies, experts at large and the delegated representatives of journals and subspecialty societies. Ultimately, 46 individuals from all six continents responded and participated in the first Delphi round; approximately half were from Europe (Fig. 1), with age and gender distribution demonstrated in Figure 2. Of these, 28 (61%) were men and 18 (39%) were women. Over half of the participants (59%) were national society representatives, and 19% were experts at large (Fig. 3). Participants were asked about their principal role, and 72% responded ‘clinical care’, with the rest distributed across clinical research, teaching and epidemiology. The secondary roles included clinical research reported by 36% and education by 24%, with some reporting bench research, administrative duties and editorial responsibilities (Fig. 4).

### Results of rounds 1–3

The results from rounds 1, 2 and 3 are shown in Table I, II and III, respectively.

**Table I:**
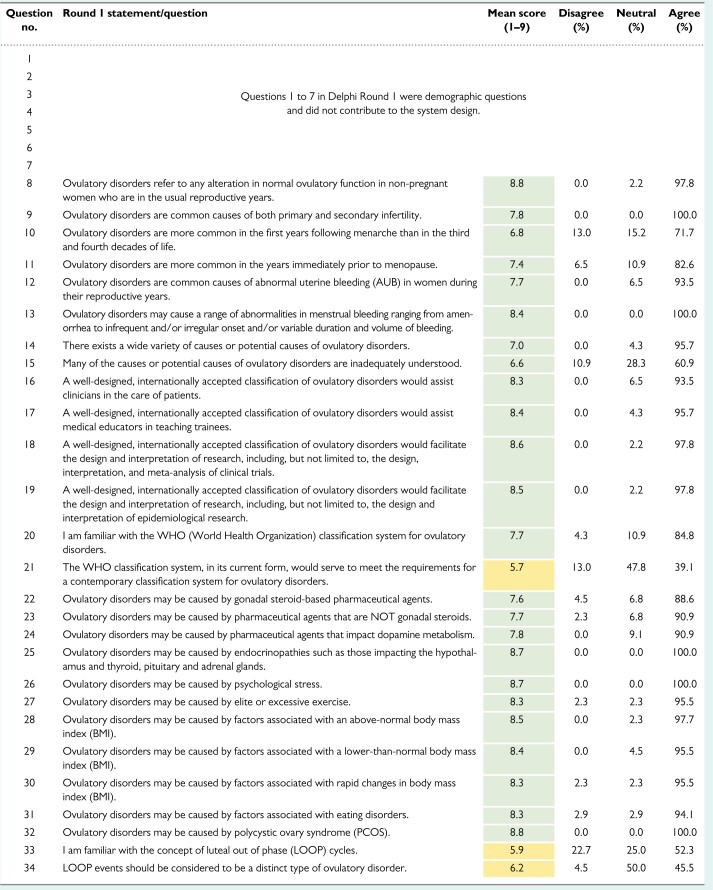

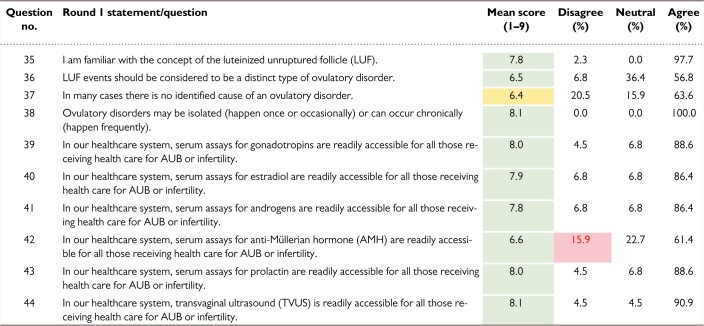
Ovulatory Disorders Classification Delphi results: Round 1.

*Note*: There were 88 invitations and 46 respondents. The first seven questions of this round were included to determine the demographics of the cohort. Questions 8–44 were designed to explore the perceived need and utility for an ovulatory disorders classification system. For agreement, a mean score of 7 (green) was required with fewer than 15% disagreeing with a statement. In this round, there was agreement on all but questions 21, 33, 34, 37 and 42, which are shaded yellow in the table. Question 42 did not reach consensus because >15% (red) disagreed with the statement.

**Table II:**
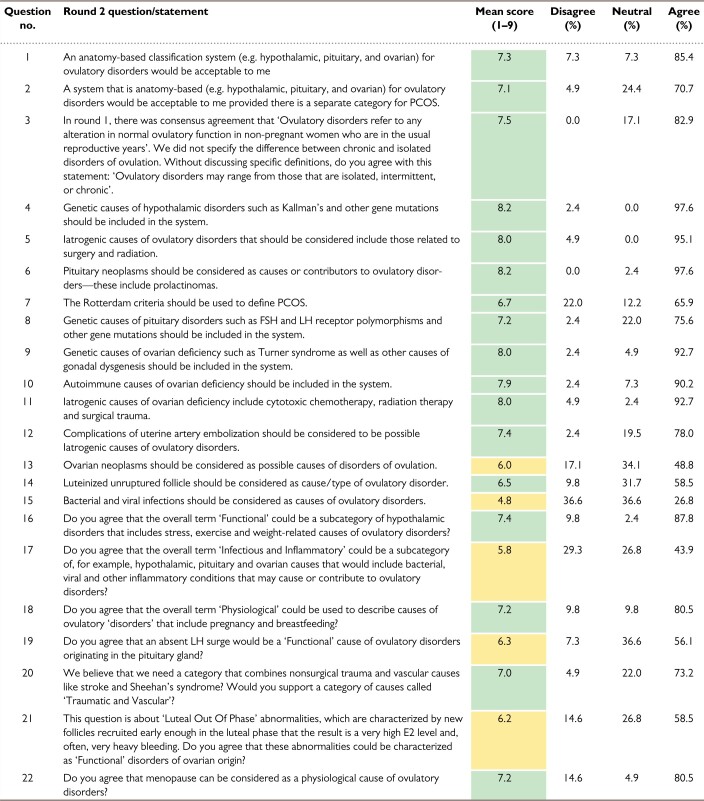
Ovulatory Disorders Classification Delphi results: Round 2.

*Note*: This 22-question round had 46 invitations and 41 respondents. Consensus (green) was obtained on statements 1–6, 8–12, 14, 16, 18, 20 and 22. The remaining statements were categorized as neutral (yellow) because there was no consensus disagreement.

PCOS, polycystic ovary syndrome.

**Table III:**
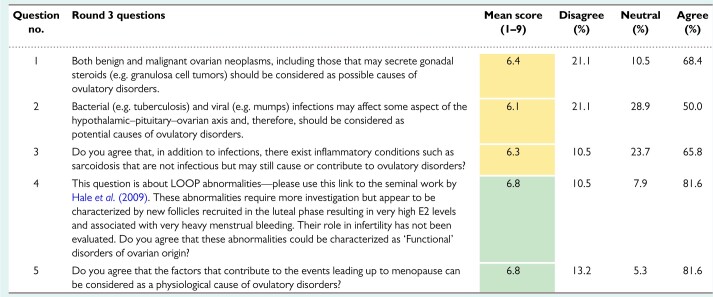
Ovulatory Disorders Classification Delphi results: Round 3.

*Note*: This was the final pre-live-meeting poll, with 46 invitations and 38 respondents. Statements 4 and 5 reached the criteria for consensus (green); statements 1–3 failed to reach consensus (yellow), but there was no consensus disagreement (mean score ≤ 3.4).

In round 1, of 37 items, there was consensus on all but five. There was general support for the stated definition of ovulatory disorders and the rationale for a consensus classification system to support research, teaching and clinical care. Respondents neither supported nor disagreed with the statement ‘The WHO classification system, in its current form, would meet the needs for a contemporary classification system for ovulatory disorders’. There was broad support for a spectrum of potential causes of ovulatory disorders except for idiopathic mechanisms and LOOP cycles (Hale *et al.*, 2009).

The ODSC took these results and developed and tested the second Delphi round before distributing it to the 46 respondents in the first round. There were 41 respondents with the results of the 22 items shown in Table II. The results of the second round suggested that there would be support for an anatomically based system (hypothalamus, pituitary, ovarian) with a separate category for PCOS. There was general support for this concept, with a mean score of 7.1. The survey also explored the notion of distinguishing chronic from isolated or intermittent ovulatory disorders, and this concept received consensus support with a mean score of 7.5 with no respondent disagreeing. Importantly, no consensus was reached on the question of using the Rotterdam Criteria (ESHRE-ASRM, 2004) to define PCOS, as 22.0% were in disagreement despite a mean overall score of 6.7. The second round was also designed to clarify some items from the first round and to identify more granular concepts relating to the pathogenesis of ovulatory disorders.

There was a lack of consensus regarding the role of ovarian neoplasms, bacterial and viral infections, and the concept of infectious or inflammatory causes in general. There was also no consensus on the role of an absent surge of LH and LOOP events. While ‘menopause’ as an etiology had a mean score otherwise sufficient to indicate agreement, 15% of the respondents disagreed, thereby preventing the attainment of consensus.

With these data, the ODSC devised a draft system based upon anatomy that included a separate component for PCOS. Before distributing to the participants, and as a prelude to the live virtual meeting of the participants in the Delphi process, a five-item third round was developed, tested and distributed. Included in the distribution to the participants was evidence describing and evaluating LOOP events and the potential role of ovarian neoplasms and infectious or inflammatory disorders in the pathogenesis of ovulatory dysfunction. Related items were modified, and the results from the 38 respondents are displayed in Table III. There was now consensus support for the inclusion of menopause and LOOP events, but lack of agreement on the role of ovarian neoplasms and infectious or other inflammatory disorders in the genesis of ovulatory dysfunction.

### Live meeting

For the live meeting, the ODSC distributed the draft system and an Excel workbook comprising a summary of the results of the three rounds and how the consensus agreements attained were integrated into the design. The live meeting was conducted on 25 August 2021, using the Zoom video platform. The meeting agenda included a review of the rationale for the process and the results of the three Delphi rounds, summarizing areas of agreement and focusing on the few places where consensus had not been reached. A total of 22 respondents could attend, so it was impossible to survey them officially. Still, there was a strong indication of support for the system based upon an in-meeting electronic poll. The formal process was the subject of the fourth round.

### Results of round 4

For this round, the ODSC sought the participants’ opinions on the draft system and tried to resolve some of the remaining items upon which there was a persisting lack of consensus. For this four-item survey, there were 39 respondents, with the results displayed in Table IV. There was support for the presented system by 95% of the respondents (mean score 8.0), with disagreement of only 2.6%. The fourth round also saw agreement that there should be a category for ovarian neoplasms. Although more than 60% supported the notion of inflammatory or infectious mechanisms, these items failed to achieve the predetermined criteria for consensus. There were some valuable comments about the specific graphical depiction of the system that will be discussed subsequently in the context of the results of the lay round.

**Table IV:**
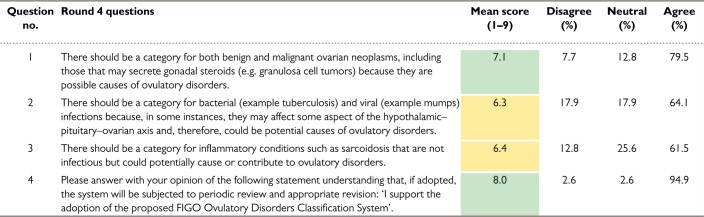
Ovulatory Disorders Classification Delphi results: Round 4.

*Note*: Delphi round 4 followed the live meeting. There were 46 invitations and 39 respondents. For agreement, a mean score of 7 was required (green) with fewer than 15% disagreeing with a statement. Here there was strong support for the system design, although there was a lack of consensus (yellow) regarding the role of infections and inflammatory conditions as contributors to the genesis of ovulatory disorders. There was now consensus support for the potential role of ovarian neoplasms as a potential cause of ovulatory disorders.

### Results of the lay round

The lay round, as planned, was conducted following the deliberations of the experts and society, and journal representatives and the development of the draft FIGO Ovulatory Disorders Classification System. The results of the 11-item survey sent to 17 individuals can be seen in Table V. The first three items were designed to obtain demographic data; all 10 respondents were women representing organizations from Africa, Europe and North America with an age distribution of 25–54 years.

**Table V:** Ovulatory Disorders Classification Delphi results: Lay round.

Question no.	Lay round statements/questions	Mean score (1–9)	Disagree (%)	Neutral (%)	Agree (%)
1	Questions 1–3 were demographic questions.				
2					
3					
4	Ovulatory disorders refer to any alteration in normal ovulatory function in non-pregnant women who are in the usual reproductive years (between the date of the first menstrual period and that of menopause).	7.2	10.0	10.0	80.0
5	Ovulatory disorders are common causes of infertility (inability to conceive spontaneously, typically for more than 12 months).	6.8	10.0	20.0	70.0
6	Ovulatory disorders are common causes of abnormal menstrual bleeding in women during their reproductive years. This means some abnormality in the frequency, regularity, duration or volume of menstrual periods—or even absent periods.	5.8	10.0	50.0	40.0
7	There are many different causes or potential causes of ovulatory disorders, and it appears that the cause is often unknown.	6.3	10.0	30.0	60.0
8	Many of the causes or potential causes of ovulatory disorders are not well understood by girls and women.	8.5	0.0	0.0	100.0
9	A well-designed system for classification of ovulatory disorders would be useful for facilitating interactions between women or patients and healthcare providers.	7.7	0.0	10.0	90.0
10	A well-designed system for classification of ovulatory disorders should improve the design and interpretation of research.	7.5	0.0	10.0	90.0
11	The system presented seems understandable and provides a platform upon which a lay audience can gain insight into the possible causes of ovulatory disorders.	4.9	44.0	22.2	33.3

*Note*: There were 11 invitations and 10 respondents. The first three items were for demographic purposes. For agreement, a mean score of 7 was required (green) with fewer than 15% disagreeing with a statement. There was a lack of consensus (yellow) regarding the potential role of ovulatory disorders in the cause of abnormal uterine bleeding as well as the notion that some causes of ovulatory disorders may be unknown. There was criticism regarding the system as presented, with a mean score of 4.9 and 44% disagreeing with the construct at that time as using language not accessible to a lay audience. These responses predated modifications in the graphical presentation of the system and the development of a patient orientation pamphlet.

There was general agreement on the definition of ovulatory disorders and their potential role in the genesis of infertility. However, there was no consensus on the contribution of ovulatory disorders to symptoms of AUB. While there was agreement that girls and women often do not understand the causes of ovulatory disorders, there was uncertainty regarding reasons unknown to healthcare providers and other medical professionals. There was a clear consensus that a well-conceived system of classifying ovulatory disorders would improve the design and interpretation of research and facilitate communication between patients and healthcare practitioners. However, the support for the draft system was mixed with a mean score of 4.9 and only 33% agreeing that the system was ‘understandable’ and one that could provide ‘a platform upon which a lay audience’ could ‘gain insight into the possible causes of ovulatory disorders’.

The comments from the participants were illuminating (Table VI) and, in some instances, mirrored comments from the other participants. Respecting these comments, the ODSC altered the graphical representation of the system without changing the content, placing the PCOS panel at the bottom, allowing for the use of the acronym ‘HyPO-P’. In addition, a draft lay version of the major elements of the system was developed with lay language that was nonetheless compatible with the medical version (Supplementary Material). This draft was distributed to lay participants and their comments were generally incorporated into the text, and into modifications of the graphical content.

**Table VI:** Lay round comments.

Technical language not accessible to all.

Lay audience do not understand medical jargon.

It is confusing that PCOS is in the left-hand column if it does not relate to any of the words in the right-hand column.

As a lay person working for a patient advocacy group, I can understand the system presented.

Would consider adding what those two columns (levels) are—anatomical/location (?), possible causes related to anatomical location. Also, would make it more clear visually which category from the right column relates to which category from the left one.

If PCOS is an exception, it’s hard to understand why it’s in this column then (if we already have a category ‘Ovarian’).

The pic is not very clear to understand by itself. It is more clear if I read the explanation at the beginning.

If PCOS is not about anatomy and stands by alone and has different causes, maybe it would be better to put it a bit separately on the pic. Because at a first glance, it looks like the causes on the right are also PCOS causes.

What I don’t personally understand is what is iatrogenic and idiopathic, and functional and how idiopathic is different from physiological. And if we speak of general audience (like women and girls) I would suggest explaining what each word means. What looks more or less understandable is endocrine, genetic, inflammatory, trauma. The rest would benefit from explanation in simple terms.

As regards structure, it’s not clear why causes are somehow grouped in three groups. Do those groups pertain to each hypothalamic, pituitary and ovarian? It looks like each group is a group of causes for each ‘organ’. Not sure what you planned to showcase.

PCOS, polycystic ovary syndrome.

*Note*: Comments reflecting the initial graphical presentation of the system. Changes in this presentation have been made without altering the actual content or design of the system.

## Proposed HyPO-P system

### Rationale and development

The system was designed to align with the results of the Delphi process (see Supplementary Table SI). There was support for a design that grouped the causes of ovulatory disorders anatomically, a logical extension of the former WHO classification but more precise and more accessible than one based primarily on hormone assays. It was, therefore, rational to design this classification system according to the levels of the hypothalamus–pituitary–ovarian axis as reflected in the second Delphi round (Table II, question 1). It was also considered essential to allow for the designation of any element that is known or suspected to alter the functionality of the organ in a fashion that could contribute to the genesis of ovulatory dysfunction, whether related to demonstrable histopathology, abnormal laboratory assays, iatrogenic mechanisms or even functional disorders without measurable laboratory features. However, it was recognized that an important cause of ovulatory disorders is PCOS since it affects 8–13% of women of reproductive age (ESHRE Capri Workshop Group, 2012). It is a complex and heterogeneous condition with comprehensive international guidelines for diagnosis, investigation and management (Fauser *et al.*, 2012; Balen *et al.*, 2016; Teede *et al.*, 2018) that cannot be confined to an ovarian origin. Therefore, it was determined that PCOS constitutes a class apart from the anatomical categorization, a notion that was supported in the second round of the Delphi process (Table II, question 2).

**Figure 5.**
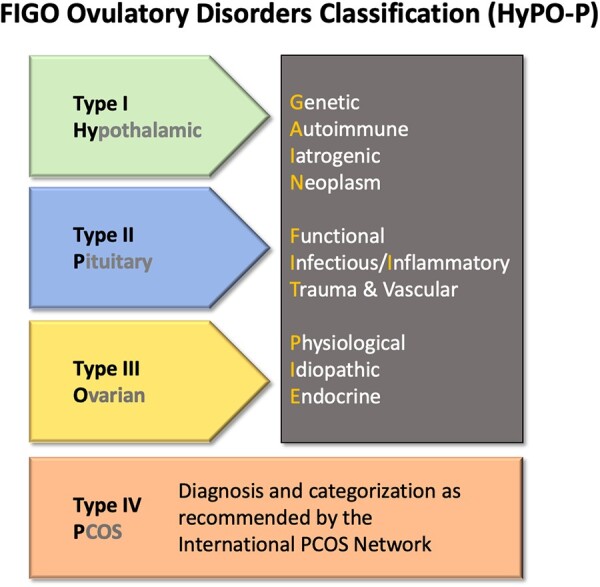
**Graphical depiction of the proposed FIGO Ovulatory Disorders Classification System.** *Note*: After the individual is diagnosed with an ovulatory disorder, the core or first level of the system is the allocation to type I, II or III disorders according to their presumed primary source: hypothalamus, pituitary gland or ovary, respectively. PCOS comprises the type IV category and the criteria proposed by WHO are to be used to determine this categorization. The second level stratifies each anatomic category (types I–III) into the known or presumed mechanism according to the ‘GAIN-FIT-PIE’ mnemonic as appropriate and applicable. FIGO, International Federation of Gynecology and Obstetrics; GAIN-FIT-PIE, Genetic, Autoimmune, Iatrogenic, Neoplasm; Functional, Infectious and Inflammatory, Trauma and Vascular; Physiological, Idiopathic, Endocrine; PCOS, polycystic ovary syndrome; WHO, World Health Organization.

Therefore, the proposed FIGO classification now includes ovulatory disorders categorized into four groups as follows: Type I: Hypothalamic; Type II: Pituitary; Type III: Ovarian; and Type IV: PCOS (Fig. 5). The system can be referred to by the acronym ‘HyPO-P’, where the ‘P’ is separated from the other three categories recognizing that it does not reside in a single anatomic location. The new system provides practical utility and a second layer, or sub-classification, for each of the three anatomically defined entitles, including discrete pathophysiological categories. These can be remembered using the acronym ‘GAIN-FIT-PIE’ (Fig. 5).

A detailed description of every known or suspected cause of ovulatory dysfunction is beyond the scope of the present paper. Still, the new classification is presented with references to some of the many included conditions. Supplementary Table SI shows the linkages between various potential causes or categories of causes and the elements in the FIGO Ovulatory Disorders Classification System.

## Use of the FIGO Ovulatory Disorders Classification System

### Clinical application

#### Identifying individuals with ovulatory disorders

The new system is designed for clinicians, educators and investigators, including those involved in basic, translational, clinical and epidemiological research. Depending on the audience, educators may focus only on the four primary categories or add the detail afforded by the second GAIN-FIT-PIE stratification.

**Figure 6.**
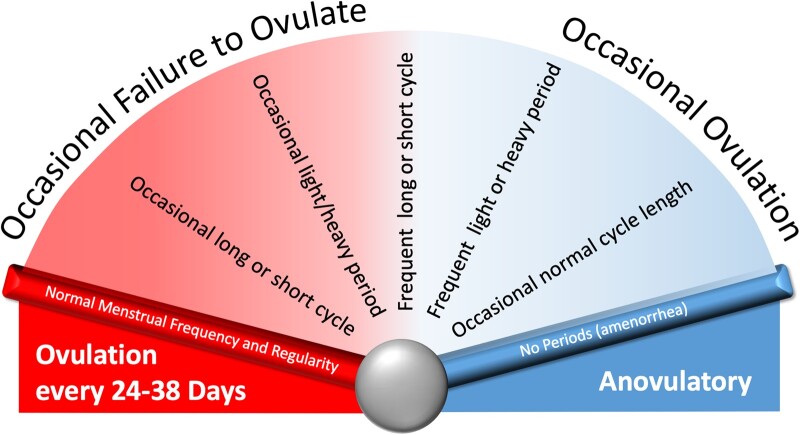
**Disorders of ovulation exist on a spectrum that ranges from occasional failure to ovulate to chronic anovulation.** *Note*: Typically, but not always these disorders manifest abnormalities in menstrual parameters, such as frequency, regularity, duration and volume of bleeding, and, in the case of chronic anovulation with amenorrhea. It is apparent that the luteinized unruptured follicle (LUF) and luteal out of phase (LOOP) disorders exist on a similar spectrum of varying frequency.

To be categorized by the system, the individual or patient must be identified as having an ovulatory disorder. Several potential clinical ‘entry points’ are based on suspicion or knowledge about the presence of an ovulatory disorder that range from delayed menarche to infrequent or irregular menstruation through to presentation with primary or secondary infertility or hirsutism or other features or findings associated with PCOS. The term ‘ovulatory disorder’ is not synonymous with the term ‘anovulation’. Instead, ovulatory disorders are considered to exist on a spectrum ranging from episodic to chronic (Fig. 6). Individuals may present with a chronic problem or may experience a singular episode where an anovulatory ‘cycle’ manifests with delayed onset of HMB. Especially in the late reproductive years, women may experience regular, predictable cycles of normal length but experience HMB as the development of follicles in the luteal phase contribute to high premenstrual estradiol levels, a process known as a LOOP cycle (Hale *et al.*, 2009).

Individuals with primary amenorrhea deserve special attention, and details regarding their investigation are beyond the scope of the present paper. However, in general, primary amenorrhea is said to be present when menstruation has not yet occurred by the age of 14 years in the absence of secondary sexual characteristics (when it is called delayed puberty) or 16 years in the presence of secondary sexual characteristics. Associated symptoms such as cyclical pelvic pain may suggest the presence of ovulation in association with a Müllerian anomaly or other obstruction that should be appropriately investigated without delay.

Most, but certainly not all, ovulatory disorders are suggested by the presence of symptoms of AUB, ranging from complete absence (amenorrhea) to infrequent or irregular onset of menstrual blood flow. Secondary amenorrhea is generally defined as the cessation of menstruation for 6 months consecutively after at least one previous spontaneous menstrual bleed (Hickey and Balen, 2003). Using data from extensive epidemiological studies, FIGO has previously determined that for those aged 18–45 years, and using the 5–95% percentiles from large-scale population studies, the normal frequency of menses is 24–38 days. Those with a cycle length of fewer than 24 days are deemed ‘frequent’ while those whose cycle length is more than 38 days, ‘infrequent’, a term designed to replace oligomenorrhea (Treloar *et al.*, 1967; Vollman, 1977; Belsey and Pinol, 1997; Fraser *et al.*, 2007a; Munro *et al.*, 2018). Even in this category, regularity varies by age; for those aged either 18–25 or 42–45 years, the difference between the shortest and longest cycle should be 9 days or less, while for those aged 26–41 years, it is 7 days or less (Munro *et al.*, 2018). Regardless, those with infrequent or irregular menstrual bleeding should be considered to have an ovulatory disorder.

Diagnosing the presence of an ovulatory disorder at the extremes of reproductive age can be challenging, depending on the perception of what is normal. For postmenarcheal girls aged under 18 years, infrequent menstrual bleeding or irregular menstrual cycles suggesting ovulatory dysfunction are common, with available evidence suggesting that the individual’s ‘normal’ cycle length may not be established until the sixth year after menarche (Flug *et al.*, 1984; WHO, 1986a,b). During this pubertal transition, ovulatory dysfunction impacts about 50% of adolescent girls in the first year after menarche with a cycle length that is typically in the range of 21–45 days (Treloar *et al.*, 1967; Vollman, 1977) but sometimes is as short as 20 days or may even exceed 60 days (WHO, 1986b). In the years after menarche, these variations change such that 6 years later, the range is similar to those of adults (WHO, 1986a,b). These issues can be explored in detail elsewhere (Diaz *et al.*, 2006; ACOG, 2015). However, it should be remembered that while common, and even ‘normal’, the individual’s experience with this transition can be disruptive at a vulnerable time in their social, psychological and physical development.

A somewhat similar experience exists at the opposite end of the reproductive age spectrum, beyond the age of 45 years, as women enter what has been called the menopausal transition, where cycle length typically becomes more infrequent or irregular before culminating in amenorrhea as ovarian secretion of estradiol declines and ultimately ceases. However, this experience is perhaps even less orderly than that of the postmenarcheal period, as there may be highly variable endocrine changes resulting in unpredictable impacts on menstrual function (Burger *et al.*, 2008). Again, what is common, and often portrayed as ‘normal’ can be highly disruptive, particularly when coupled with other symptoms.

Women who present with infertility may have accompanying menstrual symptoms typical of ovulatory disorders. However, women with cyclically normal onset of menstrual bleeding may not be ovulating, or at least not ovulating regularly, as the frequency of single-cycle anovulation in the context of normal regular cycles is in the range of 3.7–26.7% (Malcolm and Cumming, 2003; Prior *et al.*, 2015; Giviziez *et al.*, 2021). Consequently, further evaluation beyond a detailed history will be necessary to identify those with ovulatory disorders.

The optimal way to assess for ovulation and, by extension, confirm ovulatory disorders may vary according to the clinical circumstance. The menstrual history of regular, predictable cycles between 24 and 38 days remains a helpful tool, and reflects the overall experience better than evaluation of endocrine or imaging parameters from a single cycle does. While patients and clinicians have traditionally used the measurement of basal body temperature, interpretation can be difficult, so this approach should be used with caution (Bauman, 1981; Quagliarello and Arny, 1986). If available, ovulation predictor kits that measure the levels of luteinizing hormone in urine samples generally accurately reflect levels of serum luteinizing hormone and are a valuable tool for detecting ovulation in a given cycle (ASRM, 2013). Simply measuring progesterone in the predicted luteal phase may provide satisfactory evidence supporting ovulatory function, particularly when the first day of the next menstrual period is known (ASRM, 2021). Such an approach may be helpful in circumstances such as hirsutism, where the incidence of anovulation in women with cyclically predictable menstrual cycles is higher (Azziz *et al.*, 1998).

There are other, less common ovulatory disorders that may require more complex evaluation to determine if they are present in a given individual. For example, identifying LUF cycles, somewhat common in infertile women, requires both confirmation of the LH surge and the performance of serial ultrasound to demonstrate failed rupture of the dominant follicle (Qublan *et al.*, 2006). It should be remembered that scrutiny of a single cycle may not reflect the overall experience for a given individual.

#### Categorization in the FIGO Ovulatory Disorders Classification System

The new system recognizes three basic strata once an ovulatory disorder has been diagnosed. The first level is categorization by one of the four primary categories as follows: Type I: Hypothalamus; Type II: Pituitary; Type III: Ovary; and Type IV: PCOS. The second level requires assignment to the known or suspected anatomically based abnormality as directed by the GAIN-FIT-PIE acronym. The third or tertiary level identifies a specific entity causing or contributing to the ovulatory disorder. Categorizing into these levels requires that the clinician perform whatever investigations deemed appropriate to localize the site and the presumed underlying mechanism contributing to ovulatory dysfunction. For example, the individual with infrequent and irregular menses, galactorrhea, elevated prolactin and a magnetic resonance image demonstrating a pituitary tumor would categorize as a type 2—N (pituitary neoplasm). The same might be said about an individual with irregular and infrequent menstruation, mild hirsutism and sonographic evidence of at least one symmetrically enlarged ovary (≥10 ml) or an ovary with more than 20 follicles without a dominant follicle or corpus luteum, a circumstance that dictates a type 4—PCOS classification (Teede *et al.*, 2018). Use of the 20-follicle threshold is utilized only when the patient is examined with an endovaginal ultrasound transducer with a high-frequency bandwidth of at least 8 MHz (Dewailly *et al.*, 2014; Teede *et al.*, 2018).

It is recognized that the precision in determining the anatomic location and the mechanism of pathogenesis is somewhat aspirational and will vary to a degree by the disorder and the resources available to the clinician. Further discussion of the detection, characterization and management of ovulatory disorders is beyond the spectrum of the present study, which is designed to provide a structure for clinical care, investigation and education.

## Discussion and conclusion

The FIGO HyPO-P system for the classification of ovulatory disorders is submitted for consideration as a worldwide standard designed to harmonize definitions and categories in a fashion that should inform clinical care, facilitate the education of patients and trainees, and improve the ability of basic, translational, clinical and epidemiologic research to advance our knowledge of ovulatory disorders, their diagnosis and their management. The development has the general support of a broad spectrum of national and subspecialty societies, relevant journals and recognized experts in the realm of ovulatory dysfunction. The lay participants agreed with the need for classification. Their comments helped refine the graphical representation and supported the rationale for a lay-oriented explanation of ovulatory disorders presented in the context of the new system. Finally, no system should be considered permanent, so review and careful modification and revision should be carried out regularly Supplementary data.

## Authors’ roles

M.G.M.: Chair of the Ovulatory Disorders Steering Committee (ODSC); responsible for the concept, design and management of the Delphi system; management of ODSC and stakeholder meetings, compiling and analysis of data, manuscript preparation. A.H.B.: at large member of the ODSC; helped lead design and management of the Delphi process; analysis of data; responsible for converting results into the design of the system; manuscript preparation. S.H.C.: member of the ODSC; participated in the Delphi design and identification of stakeholders, and manuscript preparation. H.O.D.C.: member of the ODSC; participated in the Delphi design and identification of stakeholders, analysis of data and manuscript preparation. I.D.: Co-chair of the ODSC; participated in the Delphi design and identification of stakeholders, assisted with manuscript preparation. R.F.: member of the ODSC; participated in the Delphi design and identification of stakeholders and assisted with manuscript preparation. L.H.: member of the ODSC; participated in the Delphi design and identification of stakeholders, analysis of data and manuscript preparation. E.M.: member of the ODSC; participated in the Delphi design and identification of stakeholders, and manuscript preparation. Z.M.v.d.S.: member of the ODSC; participated in the Delphi design and identification of stakeholders, analysis of data and manuscript preparation.

## Supplementary Material

deac180_Supplementary_Data

deac180_Supplementary_Table_SI

## Figures and Tables

**Table:** 

**First name**	**Last name**	**Category**	**Representing organization/society**
Ganesh	Acharya	Journal	*Acta Obstetrica and Gynecologica Scandinavia*
Georgios	Adonakis	Expert at large	N/A
Sadiah	Ahsan	National Society	Society of Obstetricians and Gynecologists of Pakistan
Taghreed	AIhaidari	National Society	Iraqi Society of Obstetrics & Gynecology
Tengiz	Asatiani	National Society	Georgian Obstetricians and Gynecologists Association
Ricardo	Azziz	Expert at large	N/A
Adam	Balen	Steering Committee	N/A
Michela	Bedard	Lay participant	Period.org
Jennifer	Blake	National Society	Society of Obstetricians and Gynecologists of Canada
Veronica	Chamy	National Society	National Society, Obstetrics and Gynecology Chile
Ying	Cheong	National Society	Royal College of Obstetricians and Gynaecologists
Vincent Y.T.	Cheung	National Society	Obstetrical & Gynaecological Society of Hong Kong
Si Hyun	Cho	Steering Committee	N/A
Hilary	Critchley	Steering Committee	N/A
Jose Teixeira	da Silva	Expert at large	Centro de Genetica Alberto Barros
Ivonne	Diaz	Steering Committee	N/A
Colin	Duncan	Subspecialty Society	Society for Reproduction and Fertility
Amelie	Ekersley	Lay participant	Clue
Roberto	Epifanio-Malpassii	National Society	Sociedad Panamenã de Obstetricia y Ginecología
Abimbola	Famuyide	Journal	*Obstetrics & Gynecology*
Rui	Ferriani	Steering Committee	N/A
Linda	Giudice^*^	Subspecialty Society	WHO
Maargarita	Gurevich	Lay participant	Flo Health
Sioban	Harlow	Journal	*Journal of Women’s Midlife Health*
Roger	Hart	National Society	Royal Australian and New Zealand College of Obstetricians and Gynaecologists
Oskari	Heikinheimo	Expert at large	N/A
Laurie	Henry	Steering Committee	N/A
Sulaiman	Heylen	Subspecialty Society	South African Society of Reproductive Medicine and Gynaecological Endoscopy
Richard	Kennedy	Expert at large	N/A
Anna	Klepchuckova	Expert at large	N/A
Petr	Krepelka	National Society	Czech Gynecological and Obstetrical Society
Paul	le Roux	Subspecialty Society	African Federation of Fertility Societies
Kateryna	Levchenko	Lay participant	Flo Health
Dimitrios	Loutradis	National Society	Hellenic Obstetrical and Gynecological Society
Erica	Marsh^*^	Subspecialty Society	American Society for Reproductive Medicine
Noni	Martins	Lay participant	Unfertility
Raj	Mathur	Subspecialty Society	British Fertility Society
Thabo	Matsaseng	Expert at large	N/A
Rui	Miguelote	Subspecialty Society	Portuguese Society of Reproductive Medicine
Edgar	Mocanu	Steering Committee	N/A
Malcolm	Munro	Steering Committee	N/A
Eugene	Ngoga	National Society	Rwanda Society of Obstetricians and Gynecologists
Michelle	Nisolle	Journal	*Fertility Sterility*
Robert	Norman	Subspecialty Society	The Australian NHMRC Centre of Research Excellence in Women’s Health in Reproductive Life (CRE-WHiRL)
Masanori	Ono	National Society	Japan Society of Obstetrics and Gynecology
Axelle	Pintiaux	National Society	Collège Royal des Gynécologues de Langue Française Belges
Gunda	Pristauz-Telsnigg	National Society	Austrian Society for Gynecology and Obstetrics
Diana	Ramasauskaite	National Society	Lithuanian Association of Obstetricians and Gynecologists
Pernille	Ravn	National Society	Danish Society of Obstetrics and Gynecology
Jose	Reis	National Society	Portuguese Society of Gynecology
Peter	Roos	Subspecialty Society	South African Menopause Society
Irena	Rozic	Subspecialty Society	Fertility Europe
Anibal	Scarella	National Society	Chilean Society of Obstetrics and Gynecology
Katsiaryna	Sharai	Lay participant	Flo Health
Alena	Shibut	Lay participant	Flo Health
Sony	Sierra	Subspecialty Society	Canadian Fertility and Andrology Society
Anne	Steiner	Expert at large	N/A
Dominic	Stoop	Subspecialty Society	Belgian Society of Reproductive Medicine
Bettina	Toth	National Society	Austrian Society for Gynecology and Obstetrics
Zephne	Van Der Spuy	Steering Committee	N/A
Saskia	Williams	Lay participant	Infertility Awareness Association of South Africa
Lauren	Wise	Expert at large	N/A
Anusch	Yazdani	Journal	*Journal of the Royal Australian and New Zealand College of Obstetrics and Gynaecology*
Liudmila	Zhaunova	Expert at large	N/A
Meggan	Zunckel	Lay participant	Infertility Awareness Association of South Africa
Karabo	Zwane	Lay participant	Hannah You Are Not Alone

* Did not receive invite, but reviewed process and results so could report to represented organization.
